# Nursing Section

**Published:** 1904-02-13

**Authors:** 


### The Hospital.

### Nursing Section.

Contributions for this Section of "The Hospital" should be addressed to the Editor, "The Hospital" Nursing Section, 28 & 29 Southampton Street, Strand, London, W.C.

No. 907.—Vol. XXXV. *SATURDAY, FEBRUARY* 13, 1904.

### Notes on News from the Nursing World.

### THE NURSES' CO-OPERATION.

The annual meeting of the nurses of The Nurses' Co-operation has been held, and there were declared elected as nurse-representatives, Mr. J. P. Bickersteth, Miss Turner, and Miss Bath. Out of 516 voting papers sent out, only 231 were returned. The Co-operation seems in a more settled state, but the question of pensions and sick pay is agitating the nurses. Mrs. Shirley Murphy and Dr. Voelker have been re-elected members of the Committee. In future all nurses who joined before 1900 are only to pay 5 per cent. of their earnings to the office expenses. We propose to deal in full with the annual report next week.

### A STRANGE LAPSE OF MEMORY.

It will be seen in another column that the Directress of the Anglo-American Nursing Home at Rome confesses she was mistaken when she stated, as reported in The Hospital last week, in reply to questions by Lady Frederick Bruce and Miss Bulwer, that no cases of persons suffering from infectious diseases had been received in the Home during the year 1903. She now finds that early in the year one patient was admitted for German measles and that two patients who were admitted for tonsillitis afterwards developed diphtheria. She also says that, "all were taken into the Home after consultation with at least one member of the managing committee." But what can be thought of the capacity of a person for the post of matron of a nursing home who has to acknowledge that she could not remember whether cases of such a character were admitted in 1902 or 1903? And why did she attempt to rely on her memory? Surely the books of the Home show the dates of admission of every patient. If not, they ought to do so; and if they do, why were the books not consulted before the Chairman was allowed to make an erroneous statement? The incident supplies another argument in favour of the classification of the diseases. It is also a question whether the member of the Committee, whose name is not given, had any authority to assent to the admission of patients suffering from infectious diseases. According to a resolution adopted by the Managing Committee on March 21, 1902, no cases of measles, scarlet fever, or diphtheria are to be admitted to the Home, and no discretionary powers in the matter are vested in any member of the committee. Unless that resolution has been rescinded, any member of the committee who gave consent to the prohibited cases being admitted was acting *ultra vires*, and an explanation is imperatively needed, in the interests of future patients at the home.

### NURSES AND STUDENTS.

Under the heading of "Reform in Hospital Management," a letter appeared in our columns a fortnight ago to which a trenchant rejoinder is made this week. We preferred not to notice the contribution of "H" until our readers had been afforded an opportunity of criticising it. The communication of "L. H." reflects, we are sure, much more accurately than that of "H." the sentiments of sensible nurses. We believe that there are few hospitals where there are any actual regulations respecting the intercourse of nurses and students outside the institution, and though the necessity for strict limitation is not exaggerated by "L. H." it is practically only inside that the issue of hospital management arises. That a nurse would be reprimanded by a matron for merely having asked a question, or given an answer, on a professional matter to a student is very doubtful. But chatter between nurses and students on either professional or unprofessional topics in hospital would be subversive of discipline and detrimental to all concerned. A young woman who enters for training in a hospital to which a medical school is attached with the idea of talking to, or making the acquaintance of, students, is much more likely to reflect discredit on her calling than to become a good nurse, and hospital managers are wise when they take every possible precaution to thwart her designs.

### THE MIDWIVES BOARD AND THE IRISH SCHOOLS.

The last meeting of the Central Midwives Board was held on Thursday, January 28th, but the official report of the proceedings did not reach us until Thursday, February 4. We suggest that either the proceedings of the Board should be open to representatives of the press, or that the reports of them should be sent out with a little more expedition. We observe that the Board had under consideration communications from Dr. Tweedy, Master of the Rotunda Hospital, Dublin, and Professor Byers, physician to the Belfast Maternity Hospital, calling attention to the practical impossibility of pupil midwives trained at these institutions complying with the requirements of the Rules. Attention has already been drawn by us to this matter, and we expressed the hope that some means of getting over the difficulty which shuts out great Irish training schools from the benefit of the Midwives Act might be adopted. The Central Midwives Board, however, came, as they state, to a necessarily adverse decision. They regret that the suggested alterations were not brought forward before the Rules were sent to the Privy Council, as having been approved by that body it is impossible for the Board to alter them. It is certainly a pity that the authorities of the Irish hospitals did not realise the results of the Rules before they were presented to the Privy Council, but if the Board cannot move in the matter, Parliament can be asked to amend the Midwives Act.

### DISTRICT WORK FOR PROBATIONERS.

THE superintendents of the principal hospitals in Chicago have decided that in future probationers in their training schools are to have a month of district or visiting work added to their curriculum. This is an interesting departure which might be emulated with advantage in our own hospitals. It is, of course, true that in a month a hospital nurse can only gain an insight into district work. But that is just what she lacks under existing arrangements, when she receives her certificate; and an insight is all that the institution in which she enters for general training can be expected to give. Its value in the way of equipment for the pursuit of her profession does not need demonstration. Nursing in the homes of the poor is very different from nursing in a modern hospital. The experience which may be gathered, even in a month, of the special difficulties of attending the sick when they are badly housed, have none of the refinements of life, none of the conveniences to which the hospital nurse has always been accustomed, none of the aids which she has deemed indispensable, cannot fail to be useful. A month, too, is not too short a time to enable a nurse to judge whether she feels herself specially drawn towards district nursing as a career, or, in any event to learn a lesson of self-reliance which is so often, in a great degree, the secret of success.

### OVERWORK AT POPLAR AND STEPNEY SICK ASYLUM.

WE hope that on reconsideration the Local Government Board will not make their sanction of the appointment of six male nurses at the Poplar and Stepney Sick Asylum conditional upon the reduction of the female staff at present chargeable on the Metropolitan Common Poor Fund. The medical superintendent of the Asylum, replying to the proposal of the Board that the reduction should be made, shows that at present the nursing staff is totally inadequate, and that the six male nurses are absolutely needed in addition. His case rests in a nutshell; there is one nurse to about eight beds. But, in confirmation, Dr. Spurrell mentions that during the summer holidays four nurses from one of the general hospitals of London were temporarily engaged to do night nurses' duty at the Poplar and Stepney Sick Asylum. On the first night of their duty, he states, they totally failed to do their work, and presented themselves at his office the next morning, intimating that in their opinion it was an impossibility for anyone to carry out the work they had been called upon to perform. In these circumstances, to insist upon the number of female nurses being reduced, if the male nurses are appointed, would be to simply perpetuate a condition of affairs which, alike in the interests of patients and nurses ought not to be allowed to continue.

### TRAINED NURSES' ANNUITY FUND

THE twenty-ninth annual report of the Trained Nurses' Annuity Fund, which has just been issued, shows that in 1903 there was a material increase in the subscriptions and donations. In fact, as compared with 1902, they have doubled. The receipt of a legacy and the larger contributions have enabled the committee to issue an additional annuity. The report, however, says that "there are still eighteen candidates waiting their turn for annuities, some of whom were approved as far back as 1896." We are afraid that this is a case of hope deferred making the heart sick. But an organisation with a total income from contributions and investments of little over £500 a year, including a legacy of £125, cannot, in the nature of things, do much in the way of granting annuities.

### DEATH OF A MATRON.

WE regret to hear of the death of Miss Anna Margaret Rayner, matron of the principal residential home of The London Association of Nurses, who passed away at 3 Burwood Place, W., on January 31st, after a long and painful illness. Her strong religious character and steady devotion to duty had for the last quarter of a century made her an influence for good amongst the large number of nurses who came under her care.

### POOR LAW NURSES AND THE MIDWIVES ACT.

THE Farnham Guardians have received from the Local Government Board a communication respecting the proposed payment by the Guardians of the fees for registration under the Midwives Act of such of their nurses as had passed, or might in future pass, the necessary examination. The Board are willing, with regard to the latter, to entertain an application for sanctioning a gratuity in each case as it arises. As to the nurses who have already passed the examination, the Board intimate that if the Guardians propose an increase of their salaries for the present year, so as to cover the registration fee, they will not withhold their consent. The answer may be taken as a guide for the Guardians of any other Unions who are anxious to pay the fee for registration of their nurses under the Midwives Act, and it is quite satisfactory. The Board, while retaining the right to refuse a particular application for the sanction of a gratuity, are not at all likely to exercise it unless there is good reason to do so.

### ST. CATHERINE'S HOSPITAL, CAWNPORE.

As our recent announcement of the sad death of Nurse Walden from plague has provoked a good deal of interest, we may say that though plague is now abating at Cawnpore, and it is hoped that the disease will be stamped out, there are numbers of patients needing skilful nursing for divers complaints. There is one English nurse who was recently sent out, at work under the lady doctor in charge of the hospital, and there are several native nurses. The latter are not wanting in energy and devotion but they always need European supervision. The secretary of the Women's Work Section of the Society for the Propagation of the Gospel in Foreign Parts tells us that "Nurse Walden gave up everything, even life itself, in the service of others."

### THE APPOINTMENT OF A SUPERINTENDENT NURSE.

THE Christchurch Guardians have been made to understand that they cannot do as they like about the appointment of a superintendent nurse or get rid of her at a month's notice if she happens to displease them. Having reported to the Local Government Board the appointment of Miss L. E. Rogers to that position, "the appointment to be subject to termination by a month's notice on either side," the Christchurch Guardians asked for the sanction of the Board. The reply was to the effect that the Board would defer their decision for six months, before the expiration of which time they desired to be furnished with a report from the medical officer as to her fitness for the post. This is because the new superintendent nurse was not trained in a hospital or infirmary maintaining a resident physician or surgeon, the usual course pursued in such cases. The Board also called the attention of the Guardians to the provisions of the Nursing Order of 1897, by which in the event of the appointment being sanctioned, it will not be competent for the guardians to terminate it without the consent of the Board. That superintendent nurses should be able to rely upon the Local Government Board standing by them so long as they discharge their duties properly, is most essential.

### THE CHARGES AGAINST A WORKHOUSE MATRON.

As we anticipated, the matron of the Carmarthen Union Workhouse has been exonerated by the Local Government Board from the charges of drunkenness and cruelty made against her, which were mentioned in The Hospital of January 30th. But they consider that she was deserving of censure for not paying the daily visits to the female wards required from her by the regulations. The Guardians, therefore, at their last meeting, called the matron before them, and the chairman formally censured her for neglect of duty, expressing his hope that the derelictions would not occur again. The matron's rejoinder does not suggest that she was duly impressed by the censure. She rejoined "All right," and was about to state something concerning the nurse, "to clear matters up," when the chairman advised her not to say any more. Subsequently, the Guardians adopted a motion to the effect that they have confidence in the matron.

### UNWISE ADVICE AT ROCHDALE.

Hitherto, the Rochdale District Nursing Association has been conducted on the right lines. It has supplied nurses for the sick poor in their own homes, and it has not been possible for anyone to suppose that the funds collected on its behalf are supplemented by fees paid to private nurses. At the annual meeting, just held, it transpired that the Association, which was established in 1896, is for the first time in debt. This, of course, should not be the case. As the Mayor, in his speech, said, a town with over 80,000 inhabitants ought easily to find a sum of under £500 for maintaining the six nurses who are needed to pay some 20,000 visits a year to people who cannot afford to remunerate them for their services. But the managers will make a mistake if they fall back on the advice of Dr. Hayle, who, not being able to attend the meeting, wrote a letter suggesting the engagement of a special nurse for paying patients. This is the thin end of a wedge which once inserted is difficult to remove and invariably does more harm than good. There is a far better way open. The Rochdale Nursing Association appears to be admirably officered, and the fact that this year the Queen's Nurses in the town will take possession of a home of their own shows the popularity of the institution. We believe that if a determined and systematic effort is made to augment the small subscriptions, especially those of the working classes who seem so thoroughly to appreciate the ministrations of the nurses, there will be no need whatever to adopt a proposal out of harmony with the governing principles of the Victoria Jubilee Institute.

### THE SUPERINTENDENT OF CANTERBURY NURSES' INSTITUTE.

At the annual meeting of Canterbury Nurses' Institute, reference was made to the resignation of the lady superintendent. The report of the House Committee contained a warm appreciation of her work, which she is relinquishing on account of ill-health. "For over twelve years," the report says, "Miss Shaw has been most conscientious and zealous in the performance of her duties, and by her influence has nurtured and maintained such a high tone among the nurses that hardly a month passes without letters being received by the Committee from patients or their friends returning thanks for having been supplied with such skilful and pleasant nurses who have been a comfort in the house, and for whom in many cases they express friendship and affection." Another testimony to the value of Miss Shaw's work may be found in the fact that two members of the staff have already received the gratuity of £10 for twelve years' service, while two more will complete the period this year, and eleven are entitled to wear the silver badge, which denotes six years' service at the Institute. The lady superintendent of a nursing organisation, who keeps her nurses for several years, these nurses being skilful and pleasant, is the right woman in the right place.

### THE DEATH OF A PROBATIONER AT WALSALL UNION INFIRMARY.

As the result of a discussion on the part of the Walsall guardians respecting the death of Miss Carter, a probationer in the workhouse infirmary, blame was imputed to the doctors. The brother of the probationer, it is satisfactory to learn, has, on behalf of the family, repudiated this idea in the most emphatic terms. He expresses the conviction that all the potentialities of modern medical science were pressed into service for her benefit, and that in no respect were the doctors to blame. The family of the deceased probationer have also signified their appreciation of the loving and untiring services rendered to her by Sister Loach and the nursing staff.

### SHORT ITEMS.

The Incorporated Society of Trained Masseuses, 12 Buckingham Street, Strand, will hold an examination during the week commencing March 20th. Forms of application must be filled in and returned to the secretary not later than February 27th.—Sister H. F. Pocock has resigned her appointment in Queen Alexandra's Imperial Military Nursing Service.

### The Nursing Outlook.

" From magnanimity, all fear above;
From nobler recompense, above applause,
Which owes to man's short outlook all its charm."

### HEALTH VISITORS.

AMONGST the many new openings for nurses none stands second to that of health missioner in the slums of our big towns. It is a title that covers the woman sanitary inspector, but which is nevertheless somewhat less formal and more friendly; something more practical and more womanly. It is a post which requires either the National Health Society's certificate or the Local Government Board's certificate, and generally it is desired that the certificate of the London Obstetrical Society should also be held. For the health visitor is often appointed mainly to struggle against the terrible infant mortality in over-crowded neighbourhoods, and to visit and instruct the mothers how to rear their babies. Whether appointed under some public Council or some private committee, the value of the work must depend very much on the visitor herself; whether she has tact and sympathy and enthusiasm and is herself in her appearance and her behaviour an example of healthy living and a conferrer of happiness on others. It is work that particularly calls for bodily strength because it lies so much out of doors in all weathers; but it also needs initiative and imagination, for it is not work that can be laid down by rule, and it is not mechanical. It has been carried out in Manchester, York and other large towns for several years, and is about to be introduced at Battersea, Glasgow and elsewhere.

Taking York as an example, because the third annual report is just out, and was adopted only last week, we note that the health visitor is really the secretary of what is known as the "York Health and Housing Reform Association." One of the chief pieces of good work done by this association is the opening of an "Infants' Milk Depot" where sterilised milk properly adapted in three different strengths is supplied for infants at cost price. In connection with this work the secretary has in the year paid 1,476 visits to the babies and their mothers, and has as far as possible kept a record of the progress of the infants and helped the mothers in rearing and training the youngsters in the paths they should tread. In THE HOSPITAL last week there was an article on the Battersea Milk Depot, on which that at York was modelled, and it is interesting to see that Battersea returns the compliment by imitating the York health visitor. Now this lady is a late sister of the London Hospital, who subsequently got some slum experience as a settlement worker and a school nurse in Hoxton, and who also studied public health at the National Health Society. Miss Hutchinson therefore is well equipped for her work, and deserves the success which has attended it.

What it means to capture your citizen of the future while still an infant, and give him or her a fair start in life, only those nurses know who have had much experience as to the extra ordinary mixtures of food that the slum mother may try on her babe. And if we are to rear a more healthy race to follow after us, we must begin at the very beginning and see that the infants are properly fed and treated from the first. But it is not easy; the York Milk Depot does not pay its way yet, and has to be financed by one of those self-suppressing philanthropists for whom York is famous, who do so much for that city's health in housing and in other ways, but who truly are:—

"From nobler recompense, above applause."

The studying of the Battersea system of milk supply, and the over-seeing of its start in York, was in the hands of another health-worker, Miss Gertie Harlock, who was one of the first students of the special hygiene course at Bedford College. And for real practical experience of testing of water and of air, and of growing of bacteria, there is no better course for the would-be health missioner than the course at Bedford College for Women in Baker Street. But it needs a certain standard of education to enter, for the College is connected with London University, and is meant to be for educated women only who have reached the equivalent of the matriculation examination. The course takes a year—or rather runs for one session from October to the following July and includes hygiene, physiology, bacteriology, chemistry, physics and the laws of public health. The College has its own certificate, but it is as well to take also the sanitary inspector's certificate of the Local Government Board which can easily be acquired after taking the Bedford College Course. But it is well that the would-be health visitor should know her duties thoroughly, for though in certain cases she may be appointed mainly for the house-to-house visiting of mothers with infants in order to spread the knowledge of hygienic feeding, she is also often employed for inquiring into the spread of infectious and contagious complaints, for inspecting faulty sanitary arrangements, for reporting on insanitary property, even for advising on re-housing questions and perhaps for rent-collecting in model tenements—since this enables her to keep in weekly touch with all the inmates and to know fairly well what the health conditions of the district and of the people are.

It is a pity, if a nurse has the courage on leaving hospital to embark on this important and growing labour, not to be capable of rising as high as possible and of developing a new industry by her own individuality. It becomes more evident every day that it is the skilled and trained worker who is wanted to deal with those big problems which our civic authorities find so trying. And though the solving of these perplexities must be largely left with the framers of our country's laws, still the health missioner has the eyes that see the actual evils, and if she has a well-trained brain and a good store of knowledge, she can accumulate the facts on which future legislation must be framed. Indeed, the importance of the work to be done must not be lost sight of, for we are coming to recognise how much health and righteousness depend on one another, and how the saving of bodies leads also to the saving of souls. Women were never backward as missionaries to the heathen of all lands; let them never be backward in becoming health missioners to those of this country, who, by reason of their environment, are handicapped both as regards bodily and spiritual development.

### Lectures Upon the Nursing of Mental Diseases.

By ROBERT JONES, M.D.Lond., B.S., F.R.C.S.Eng., M.R.C.P.Lond., Resident Physician and Superintendent of the London County Asylum, Claybury.

### LECTURE V.

It is presumed that those for whom these lectures are prepared have a satisfactory acquaintance with elementary anatomy, and we shall not describe in any detail the structure and relation of the organs connected with the nutrition of the body or the building up of its tissues, but shall refer only to the physiological processes involved, these being the circulation of the blood, the respiration, and digestion. In regard to these activities a simple but an accurate knowledge is essential.

*Circulation of the Blood.*—This is very greatly, indeed we may almost say completely, under the control of the nervous system, for through the brain the heart can be made to beat quickly or slowly, it can get relief from overstrain by the action of its own centre in the medulla oblongata, and each organ can thus obtain the blood it requires during activity, or when at rest. Two nerves called the *vagus* nerves—one on each side—run down the neck and supply the lungs, heart and stomach. They arise in a centre in the medulla oblongata which is quite close to another, the vaso-motor centre, to which we shall presently refer. The vagus nerves contain nerve fibres—the *inhibitory*—which cause the heart to beat slower when the needs of the body require it. From the sympathetic ganglia, mentioned in the first lecture, and which are situated at the root of the neck, another set of nerves enter the heart and cause it to beat faster and stronger. These are called *acceleratory* or *augmentary* fibres. They arise in the medulla and run down in the spinal cord, emerging in the thoracic portion with the anterior roots of the spinal nerves. In addition to these two kinds of *efferent* nerve fibres to the heart, there arise also in the heart a series of *afferent* fibres which travel up the vagus nerves to the vaso-motor centre in the medulla. A stimulus passing from the heart along these fibres to this centre can cause the small arteries all over the body to dilate, and by thus lowering the blood pressure, the heart can expel the blood with less effort, so that when feeling strained it can get relief. We see now that the controlling of the circulation through the nervous system may occur in two ways, viz., by a regulating action upon the heart itself—the central and chief organ of the circulation—and also upon the distant organs of the circulation, viz., the blood-vessels. The fine nerves known as the vaso-motor, controlled by the centre in the medulla already referred to, end in a network over the arteries. There are two kinds of vaso-motor nerve fibres, called *vaso-dilator* and *vaso-constrictor*; the latter are always acting to keep up the tone of the blood-vessels, that is, to keep them always in a state of tonic contraction. They pass to the sympathetic ganglia, which lie on each side along the front of the vertebral column, and then out from these ganglia over the arteries. The *vaso-dilator* fibres run with the ordinary nerves (cranial and spinal), and from these are likewise distributed over the walls of the arteries. The vaso-motor centre in some way continually and constantly keeps the vaso-constrictor fibres acting, so that the brain, through the constriction of the small arteries of the body, is well supplied with blood. When the vaso-motor centre in the brain is destroyed the vaso-constrictor fibres are paralysed, all the small arteries of the body dilate, and there is scarcely any blood pressure in the arteries. There are many mental conditions which affect or disturb the vaso-motor mechanism; blushing is an example of vaso-dilatation. Fear produces pallor and constriction of the blood-vessels. In epilepsy and general paralysis of the insane there is probably a disturbance of the vaso-motor apparatus.

The circulation of the blood takes under two minutes to complete a whole circuit of its course, although some of the blood—such as that in the middle of the tube of the blood-vessel—completes the circuit in 20 seconds, so that a blood corpuscle is said to be able to pass over the whole circuit in that time. The blood flows faster in the narrower channels as in the large arteries, but as these divide and spread out into other smaller arteries and capillaries the flow is slower—the channel being wider. In the aorta the flow is at the rate of about 15 inches in a second, in the capillaries it moves not more than about one-sixteenth of an inch—the size of a pin's head—in the same time, whereas in the large veins the blood moves 10 inches in a second. The united area of the capillaries arising from an artery is much greater than the contents of the artery they spring from, or the vein they collect into, hence the flow is slowest in the capillaries.

The pulse is the wave from the distention and the contraction of the artery caused by the expulsion of blood at each contraction of the heart. This wave travels much more rapidly than the blood; it is estimated to be 18 times as fast. The contraction of the heart is called the *systole*; the period of rest or pause during which the heart is refilling is called the *diastole.* The heart alternately works and rests. The pulse indicates whether the heart-beat is strong or weak, regular or irregular, frequent or infrequent, and it thus forms an important guide to the condition of the heart. The pulse normally beats about 70 to 80 times a minute. In the new-born child the beats are about 120 to 140 a minute, this rate gradually diminishes up to the thirtieth year of age, and then rises somewhat slightly again in old age. The heart, as we know, is completely divided into two halves by a muscular partition, and there is no communication between these except in a roundabout way through the lungs. The cavities of the heart are four, each side being divided into two—a receiving and a discharging cavity. The auricles receive and the ventricles discharge the blood. These cavities are separated from each other by openings guarded by valves, the *tricuspid* on the right side and the *mitral* on the left, and there are also valves, called *semi-lunar*, separating the ventricles from the arteries—the pulmonary artery and aorta—which convey the blood from the ventricles to the lungs and the body. When these valves are diseased very serious and definite symptoms are produced. The blood makes two circular tours, one through the body generally and the other through the lungs. The size of the auricles when at rest is a little smaller than the ventricles, but when fully distended—and they are more elastic than the ventricles—the cavities of both are about equal. The left ventricle having to drive the pure aerated blood over the body, has three times as much work to do as the right, which only drives the blood through the lungs. The pressure used by the left ventricle to drive the blood would raise a column of water to a height of 5 feet, and at each contraction about 3 oz. of blood are driven out of the ventricles. The two ventricles fill together and empty together in perfect rhythm, working day and night, contracting and resting alternately, and in this way keeping up the circulation of the blood. The arteries and the veins are the conducting tubes, the capillaries are the distributing apparatus, and where the greatest activity prevails there the capillary network is most developed, as in the brain, the muscles, and the glands. The movements of the heart are quickened by heat, by excitement, by muscular action during exercise or excitement, such as that of mania, also under the influence of fear or other painful emotions. The rate of the pulse is slower in some mental states, such as that of melancholia, and in the condition described as stuporose insanity.

If serious damage or injury is done to any part of the body the circulation will cease therein, because an alteration has taken place in the capillary wall, and the corpuscles will stick to it, thus impeding the flow of blood. When the circulation stops in this part, the tissue dies, and can only be removed through the action of the white corpuscles, which, as stated, ooze through and creep in from the capillaries of some neighbouring parts. The circulation can be stopped artificially, as by tying something tight round a limb. This is how hæmorrhage is stopped in a severed artery, pressure being applied above the wound, that is, nearer to the heart. The blood flows in jerks from a wounded artery. If hæmorrhage occur from a wounded vein the flow is steady. The blood in the veins circulates mainly by the contractions of the heart, in part also by muscular movements, and especially by those connected with respiration. The ligature, in hæmorrhage from a vein, should be applied below the wound and farthest from the heart. It is important in all cases of hæmorrhage to apply direct pressure first, against a bone if possible, and not to attempt indirect pressure unless and until this has failed. It is of signal necessity that the nurse attending upon a mental case should thoroughly understand the various methods of controlling hæmorrhage in the different arteries, as the insane not infrequently make attempts at suicide by cutting the blood-vessels, and the nurse should always be on the alert in cases of mental depression to prevent the possibility of this, and knives, scissors, or sharp cutting instruments should always be carefully guarded or put away, for the means to do ill deeds may make ill deeds done.

### A Nursing Experience on Mount Lebanon.

BY AN OCCASIONAL CORRESPONDENT.

There are probably not many nurses whose honest endeavours to fulfil their duties have led to their excommunication, but the fact that I have undergone this ordeal and that as yet, though it took place eight months ago, no dire fate has overtaken me, may be of interest to unexcommunicated sisters. I was taking temporary charge of a small hospital of 20 beds—the "Friends'" Mission Hospital —in Brumana, a beautifully-situated village some four hours' drive up from Beyrout, and was the only trained nurse on that side of the Lebanon. Here the Syrians are very reluctant to come into the hospital if they are seriously ill, having a great horror of dying away from home, consequently the natives whom we try to train as nurses have very little practical experience of critical cases. In these circumstances the desirability of nursing the patients in their own homes, for their own sake and that of our nurses, prompted us to attempt, when possible, a sort of district nursing, though with such a limited staff it usually ended in my going alone if the case were too critical to trust to an inexperienced probationer, and the hospital too full to spare a worker as well as myself.

### My Priestly Patient.

Thus it came about that I quartered myself in a little primitive village some three miles from Brumana, to nurse an old Maronite priest suffering from double pneumonia. A priest in a small village in Syria is not as our clergy, though he himself must have a certain amount of education so as to read both Arabic and Syriac—the ancient language, popularly supposed to be that of Adam and Abraham, and therefore used in a great part of the services—in many cases his family remain unlettered peasants, his sons are frequently humble tillers of the soil, his wife and daughters working, when their domestic duties are over, to add a little to the family exchequer, and all of them helping in the great labour of the year—the rearing of silkworms in April and early May. It is worth noting that among the Maronites a married man may enter the priesthood, but an unmarried man taking holy orders must remain a celibate.

### The Sick-Room.

So the house I came to was but a peasant home, the inner room having its mattress on one corner of the floor, where the patient—who had been ill some six or seven days—was getting what rest he could with all his priestly garments still on, mitre-like hat included, in an atmosphere fœtid with the breath of scores of visitors, who trooped in and out (apparently mostly in!) all day long to offer their sympathy and salutations. After the inevitable Arabic greetings were exchanged, even if "Houry Yusef" (Priest Joseph) was too weak and weary to enunciate the conventional answer "God's peace to you! Well. Praise God!" the callers would all sit round the bed, the more honoured ones on any available chairs, the lesser lights—including the women—on the floor, gazing at the "afflicted of God," sighing, probably recounting the sufferings of a father or an uncle who seemed just so before he died, or audibly wondering how long "our father" would yet be spared to them, and pitying themselves in the sorrow that was about to overshadow them. Of course, being a priest, and so a public character, my patient had more than the usual number of visitors, and even a stony-hearted English nurse finds it hard to forbid a sight of the dear invalid to men—and sometimes women—who have tramped for hours over rough roads after their work is done in order to salute their "father." Here in the East the sick-room of Jairus' little daughter is often brought vividly to my mind!

### Clearing the Room.

To kindly and good temperedly eject some twenty visitors—eighteen of whom declared that they at least had a right to stay, being a brother, an uncle, a sister's husband's brother, or a son of his wife's maternal aunt—was a work of some time, particularly when the ejector's Arabic vocabulary amounted to some thirty words! Severity is not permissible; to offend them would be to defeat one's own ends, but at last the room was comparatively clear, a window was opened, and it became possible to breathe, and to investigate more closely the patient's condition, and the nursing resources—the latter extremely limited.

### A Hint to Nurses.

In case any reader should find herself similarly placed, I may say that perhaps the most effective method to gain one's end is to hint at infection, particularly at close quarters and without ample fresh air—this usually leaves only the nearest and dearest in the room. There is an utter lack of scientific knowledge among these ignorantly superstitious people. They go on their own lines, and if the patient dies it is "the will of God," but if a doctor—often not consulted until the eleventh hour, when the case is all but hopeless—tries some, to them, novel treatment unavailingly, it is the doctor who has killed the patient. So one must exercise great tact and patience, and the light of modern science must be let in just as gently as the pure outside air which has been rigidly excluded in accordance with the popular theory that vitiated lungs must require vitiated air. The struggle to keep the indefatigable kind inquirers outside was a strain far more trying than the nursing, and I was thankful to welcome night, though with it came delirium and the difficulty of diplomatically controlling such a patient without knowing his language. There was one girl, of whom the village was justly proud, who spoke French, and who had been brought to translate when I was in difficulties, but I had, of course, sent her home to bed. Finally, exhaustion overcame delirium, and the early morning brought a fall of temperature and consequent rest.

### Another Caller.

The next day I still fought with would-be visitors, the priest's eldest son supporting my efforts, but his wife, daughter, and sisters were useless, though willing enough to do the little they could. Late in the evening came a younger priest from miles away down in the plain. I afterwards learnt he was the bishop's secretary and was sent with authority, but at the time, not understanding half that was told me, I merely noted that he evidently considered himself of great importance and was arrogant in the extreme. Of course, he expected to come in, and equally, of course, I expected him not to do so, looking to him to understand and aid us all the more because he was a man of some education. I tried French, and finding that he spoke it well, took pains to explain to him the situation. My patient was evidently at that time entering the crisis, sleeping fitfully but heavily, perspiring very freely, the temperature dropping, and the pulse very weak and irregular. Knowing that this priestly visit was not a religious one, all necessary rites, even to extreme unction, having been already performed, I explained to the newcomer that if he disturbed our patient just then he must be entirely responsible for the consequences, while on the other hand if he would be good enough to help by example, and by translating the situation to others, it would be of immense benefit. He very stiffly agreed with me, and I returned relieved to the inner room.

### The Worst Happened.

Three minutes later the young priest entered, and, not attempting to conceal my surprise, I begged him in an undertone on no account to awaken the patient at this critical juncture, but just to take his look as quietly as possible. Ignoring me, he approached Houry Yusef, calling him loudly by name! I felt too disgusted to remonstrate further, but began putting on my outdoor uniform with much show of disapproval. This action at once collected round me the invalid's relatives, clamouring in an unintelligible babel, undoing my buttons as fast as I fastened them, removing my bonnet, closing the door and guarding it so that I could not leave. Their actions were as graphic as mine, and quite as unmistakable. In vain I begged them at least to let us talk outside the sick-room, or more quietly; the hubbub continued until the lordly cause of it all condescended to join the fray and denounce me violently in French, declaring he would excommunicate me, the doctor, his medicines, and all Maronites who dared to avail themselves of one or the other. His last denunciation may have had some point, but the first three were so ludicrous that they failed to be duly impressive; in fact, had it not been for the possible tragedy playing itself out on the bed in the corner, the scene would have been superlatively comic—a torrent of vehement French poured at me from one side, seven or eight minor streams of gesticulatory Arabic from the other, one side having no comprehension of the other beyond grasping the fact that it was by no means peaceable, while I, the unwilling centre of this uproar, could not translate to them though I would, and my denunciator would not though he could.

### Final Satisfaction.

To my intense surprise and joy the old priest slept through it all undisturbed—and subsequently through his crisis—and in a day or two I was able to return to my hospital, a blighted excommunicant, but comforted in this extremity by the great satisfaction of knowing my patient was, humanly speaking, out of danger.

### The Central Midwives Board.

A meeting of the Central Midwives Board, Dr. F. H. Champneys in the chair, was held at the Board-room, 6 Suffolk Street, S W., on January 28th.

On the recommendation of the Standing Committee the Board adopted three sets of questions to be addressed respectively to those applying for approval or recognition in the following capacities:—(*a*) Institutions applying for approval of their certificate, or for recognition as approved institutions under Section C. 1 of the Rules. (*b*) Registered medical practitioners seeking recognition as teachers under Section C. 1 (3). (*c*) Certified midwives applying to be approved for the purpose of signing Forms III. and IV. under Section C. 1 (2).

Applications for recognition as approved institutions under Section C. 1 of the Rules were granted to the National Maternity Hospital, Dublin; Edinburgh Royal Maternity and Simpson Memorial Hospital; and the City of London Lying-in Hospital.

### The Irish Difficulty.

A letter was read from Dr. E. Hastings Twedy, the Master of the Rotunda Hospital, Dublin, calling the attention of the Board to the practical impossibility of pupil midwives trained in the Rotunda complying with the requirements of Section C. Rule 1. Sub-section (1). Personal conduction of 20 cases, and sub-section (2), 10 days' puerperium. It was hoped that the curriculum of the Rotunda might be accepted as an equivalent to the course of training proscribed by the Rules, or that such exception or modification might be made as would enable the Rotunda nurses to qualify for the Board's examination.

After consideration of the subject, it was unanimously resolved, "That having considered the letter addressed to them by the Master of the Rotunda Hospital, the Board regret that the suggested alterations were not brought to their notice before the Rules were sent to the Privy Council, as having been approved by that body, it is impossible for for the Board to alter them."

A letter of similar purport was read from Professor Byers, Physician to the Incorporated Belfast Maternity Hospital, and a copy of the foregoing resolution was ordered to be sent in reply.

### A Thousand Additions to the Roll.

After consideration of applications for certificates the names of 1,040 women were passed under Section 2 of the Act, and ordered for entry on the Roll. Of this total 269 claimed as holding the certificate of the Obstetrical Society of London, 14 that of the Rotunda Hospital, Dublin, 25 that of Queen Charlotte's Lying-in Hospital, 14 that of the Glasgow Maternity Hospital, 14 that of St. Mary's Hospital, Manchester, two that of the Liverpool Lying-in Hospital, one that of the Edinburgh Royal Maternity Hospital, one that of the City of London Lying-in Hospital, and 699 were admitted as having been in bona fide practice for one year prior to July 31st, 1902.

### The Anglo-American Nursing Home at Rome.

THE following circular has been issued to the subscribers to the Anglo-American Nursing Home at Rome :—

I beg to call your attention to the accompanying letter in rectification of an erroneous statement the Chairman of the meeting of January 27, 1904, was unintentionally led to make.

For the managing committee S. N. VANSITTART,
Rome, February 5, 1904. Hon. Secretary.

TO THE CHAIRMAN OF THE GENERAL MEETING OF JANUARY 27, 1904.

SIR,—I have discovered by reference to the register of patients that I made a wrong statement at the general meeting on Wednesday when I said that no persons suffering from infectious disease had been admitted to the Home during the year 1903.

I find that early in that year one patient was admitted for German measles and that two patients who were admitted for tonsillitis afterwards developed diphtheria. The three proved mild cases and were discharged cured after a short period. All were taken into the Home after consultation with at least one member of the managing committee. Without the register I was uncertain when appealed to, as to the exact dates of these admissions, but after a moment's hesitation I was firmly convinced they belonged to the season of 1902 rather than to the season of 1903.

This was a mistake for which I desire to express my regret.

I am, yours faithfully,
C. KIRKPATRICK,
Rome, January 31, 1904. Directress.

### Everybody's Opinion.

[Correspondence on all subjects is invited, but we cannot in any way be responsible for the opinions expressed by our correspondents. No communication can be entertained if the name and address of the correspondent are not given as a guarantee of good faith, but not necessarily for publication. All correspondents should write on one side of the paper only.]

### THE ANGLO-AMERICAN NURSING HOME AT ROME.

MISS M. SEDGWICK writes: As one of the general public I attended the meeting of the Anglo-American Nursing Home held on January 27, and as a trained nurse I failed to see that any objection could be raised to a list of the diseases treated in the Home being given if the patients' names were not stated. The disputed points seemed no longer the unnecessary discomfort of the nurses, or the neglect of the doctor's orders by the matron, but the determination of the committee to have its own way regardless alike of patients, nurses, or doctors.

### REFORM IN HOSPITAL MANAGEMENT.

"L. H." writes: I hope that "H." is not a nurse. She writes to protest against nurses being forbidden to speak to students in the hospital, and even if not allowed to do so in the hospital, she protests against the same rule applying outside, and asks that nurses should be allowed the same freedom that other women have. She says that to her it seems that, in this respect, a nurse has not as much liberty as a school-girl. I cannot help wondering at what a curious school "H." must have been brought up, if such be her experience. Nurses are not of the servant class who can "keep company" without any divergence from what is proper and usual. "H." must surely know that no girl of respectable bringing up is allowed to tour about London with a young man. Would she advocate that every nurse should be allowed to make arrangements with students to spend their time off duty with them? Where does she think this would end? How nice it would look to see rows of students waiting outside the gates for the nurses to come off duty! Does she think that the best nurses would do this, and if not the best, does she think a proper tone could be preserved in a hospital with its worst nurses on intimate terms not with, probably, the best students? Would such friendly relationships conduce to good work in the wards or would it conduce to students visiting the wards where their "young woman" is? The idea is atrocious. If "H." is working in a hospital, does she not know exactly which nurses are the ones who "run after the men," who are too free and easy with them? Are they the best ones? Can she say that in a ward where such nurses are, or where the sister is slack about this sort of discipline, the patients do no suffer? My experience is that they do. I would wish a nurse to have the liberty any respectable and well brought up young woman has. I should wish them to have the same liberty which my own daughters have. Nature is nature, and if a mutual attraction springs up between nurse and student the proper way of meeting is at either his home or hers, or at the home of some acquaintance of one or the other. I can hardly write patiently of a nurse who thinks that one of the objects or amusements of hospital life is the talking to students in the hospital and the meeting them outside. But I hope "H." is not a nurse and not even a woman.

### ENEMA RASHES.

"A SISTER" writes: Lately it has come more to my notice that rashes of an urticarial nature often come out over a patient the day after an enema has been given. Of course, I know that turpentine when given in an enema often causes a rash, but these cases that I have noticed lately have been after giving an ordinary enema of soap and water. I thought at first that perhaps it was due to the using of common yellow soap, but as lately I have used only pure soft soap such is not the case. It does not seem quite so prevalent in adults as in children. I should be glad to know if others could tell me if they have also noticed this and would like to hear their opinions.

[Rashes may occur, especially in children, after ordinary soap and water enemas, as well as other kinds of injection. —ED. THE HOSPITAL.]

### ASSISTANT NURSES IN WORKHOUSE INFIRMARIES.

"AN ASSISTANT NURSE IN THE NORTH" writes: Will you please allow an assistant nurse to thank the matron who kindly took up the cudgels on our behalf in your last week's issue. We deeply feel our insecurity in the matter of a three yearly certificate. Although trained in a major training school (for less than three years) and then serving as a nurse in a workhouse infirmary for several more years, we are debarred from taking a higher position. Some of us have worked side by side with the termed "fully-qualified nurse," and have felt surprised at their incompetency. Nothing in a nurse's training will instruct her as to management of the smaller infirmaries except practical experience. I venture to say that many assistant nurses would pass the theoretical examinations of the three-yearly nurse, with credit, but they do not have a chance to do so. They apparently can pass the examination for the L O.S. certificate, and this they are allowed to try for. It seems as if the woman herself does not count in sick nursing, but only her three-yearly certificate. We quite see the advisability of a year's or two training, preferably at a major training school, as nowhere else can the discipline of hospital life be obtained. But why those who have had this and have served boards of guardians creditably for some years, should always be labelled "second grade nurses," and never given a chance to rise above the same, we fail to understand.

### AN ALTERNATIVE TO STATE REGISTRATION.

"A NURSE" writes: Definite arrangement under which there would be secured to the public that skilful and sympathetic help which is needful for a patient requiring to be "nursed," is, of course, much to be desired, and should be the object of those whose hearts and heads are truly engaged in the matter. But it cannot be granted that "State registration"

is the appropriate way of attaining that object. The competency of nurses or nursing is no more part or province of the duty of the State, as such, than is the efficiency of any individual professional man, or any individual trader. State registration would not elevate or maintain the elevation of the status of nurses. The proper mode, under the better public knowledge and experience that now exists, is that there should be established, either by Royal Charter or by Act of Parliament (preferably by the former), a university to be called, say, "The University of Medical Nurses." This university should be founded on a basis analogous to that of the University of London, with affiliated colleges or branches over the United Kingdom of Great Britain and Ireland. The existing hospitals might be used, under proper arrangements with the several governing bodies thereof for the formation of the affiliated branches. Everyone obtaining the diploma of the university should be entitled to the degree of M.N., *i.e.*, medical nurse, and there might well be an advanced degree D.N., *i.e.*, Director and Directress of Nurses. Members of the British Nurses' Association and of other associations considered of sufficient standing should be on request admitted to the degree of M.N., or in proper cases of D.N.

### TREATMENT BY COLLIERS' WIVES.

"The Matron of a Cottage Hospital" writes: It is curious, in these days of rapidly advancing education, to come across such extreme ignorance as occasionally prevails in some of the colliery districts of South Wales. Here are particulars of three well-authenticated cases of "treatment" as understood by the colliers' wives. The first was that of a woman suffering from gastric irritation. Inquiry elicited the fact that she had regularly been in the habit of swallowing a woodlouse, whenever she felt at all out of sorts. The second was that of another woman, who, when told that she must get some leeches for her husband, did as the doctor had ordered, but on her return from the chemist's, instead of asking for further instructions, she fried the leeches and gave them to the unfortunate man to eat. These two cases are vouched for by the medical man who attended in each instance. The third came under my own observation. A patient arrived at the hospital suffering from "a bad leg" When the bandages were removed a curious sticky-looking mass was revealed, which proved to be a poultice of boiled snails, which had been applied to the varicose ulcer by a well-meaning neighbour.

### Novelties for Nurses.

By Our Shopping Correspondent.

### "NIGHTINGALE" COLLARS AND CUFFS.

The "Nightingale" collars and cuffs, to be procured from Messrs. C. E. Mey and Co., 116 Newgate Street, E.C. especially recommend themselves to the nurses of infectious cases, and to private nurses who are so frequently moving from place to place. They are made of a paper substance with a thin linen facing. They look most neat and substantial. They can be obtained for less than the cost of washing collars and cuffs, and they last clean longer, so they are both economical and convenient.

### Where to Go.

In Aid of the London Hospital.—Miss Maree Ainslie has arranged a concert in the Queen's Hall on Wednesday, February 24th, at 8 p.m. Many well-known artists have promised their assistance; "The Follies" will appear and the band of his Majesty's Irish Guards will take part in the concert. Tickets may be obtained at the Queen's Hall.

Anderson and Anderson's Sale, 37 Queen Victoria Street.—This firm are now holding a sale of indiarubber and waterproof goods. Their commodities are of the highest excellence and this is an opportunity of securing the best articles for the price for which very inferior ones are usually bought. There is a special sale catalogue ready for applicants.

### Appointments.

Addenbrooke's Hospital, Cambridge.—Miss E. E. Baldry has been appointed night superintendent and Miss Evelyn Ward sister. Miss Baldry was trained at the London Hospital, where she afterwards became staff nurse. She served for two years as army reserve sister in South Africa and holds the L.O.S. certificate. Miss Ward was trained at Addenbrooke's Hospital, Cambridge, where she afterwards became staff nurse. She has also held an appointment at the Poplar Hospital, London.

Cottage Hospital, Dartmouth.—Miss Edith Blackler has been appointed matron. She was trained at Taunton and Somerset Hospital, Taunton, and has since been staff nurse at the Rous Memorial Hospital, Newmarket, charge nurse at the Park Hospital, Hither Green, and staff nurse at the District Hospital, Yeovil.

Fever Hospital, Maybrick Hill, Liverpool.—Miss M. Corkill has been appointed charge nurse. She was trained at Blackpool General Hospital, and has since been staff nurse at Newport County Hospital, Monmouthshire and the City Hospital, Grafton Street, Liverpool.

Leavesden Sick Asylum, King's Langley, Herts.—Miss Florence M. Boyce has been appointed superintendent and demonstrator to the nursing staff. She was trained at the North London Hospital for Consumption and Diseases of the Chest, and also at the Mile End Infirmary, where she was staff nurse for a year. She has since been attached to the All Saints' Nursing Institution, and has done private nursing. She has also been engaged as lecturer on nursing, sanitation, and hygiene to the London School Board. She has recently held the post as sister in charge of the new annexes, Tooting Home, S.W. She holds the L.O.S. certificate.

Lowestoft General Hospital.—Miss M. O. Cunning has been appointed senior charge nurse of medical and surgical wards and theatre. She was trained at the Liverpool Hahnemann Hospital, and has since been staff nurse at Newport County Hospital, theatre nurse at the Children's Infirmary, Liverpool, and charge nurse at the City Hospital, Grafton Street, Liverpool.

North Argyllshire Nursing Association.—Miss C. M. Nicol has been appointed district nurse for Tobermory. She was trained at Leith Hospital and at Belvidere Fever Hospital, Glasgow, and has since been charge nurse at the Eastern Hospital, Homerton, charge nurse at the Northern Hospital, Winchmore Hill, charge nurse at the Park Hospital, Hither Green, charge nurse at the Maryfield Hospital, Dundee, and district nurse for Laurencekirk, Kincardineshire

Stapleton Union Hospital.—Miss Florence A. Kemp has been appointed staff nurse. She was trained at Fishponds Hospital, Bristol. She holds the L.O.S. certificate.

Victoria Cottage Hospital, Woking.—Miss Evelyn Hurlbatt has been appointed matron. She was trained at Guy's Hospital, London, where she has since taken sister's holiday duty. She has also been matron of the Reynard Cottage Hospital, Willingham, Gainsborough, matron of Kendal Hospital, and lady superintendent of a surgical home at Norwich.

### To Nurses.

**We invite contributions from any of our readers, and shall be glad to pay for "Notes on News from the Nursing World," or for articles describing nursing experiences, or dealing with any nursing question from an original point of view. The minimum payment for contributions is 5s., but we welcome interesting contributions of a column, or a page, in length. It may be added that notices of appointments, entertainments, presentations, and deaths are not paid for, but that we are always glad to receive them. All rejected manuscripts are returned in due course, and all payments for manuscripts used are made as early as possible after the beginning of each quarter.**

### Echoes from the Outside World.

### The Royal Wedding.

THE King and Queen left London on Tuesday for Windsor, in order to be present at the marriage between Princess Alice of Albany and Prince Alexander of Teck, which took place in St. George's Chapel at 12.30 on Wednesday, the anniversary of Queen Victoria's wedding day. In addition to their Majesties, the Prince and Princess of Wales, and the members of our own Royal family, the illustrious company who assembled to witness the ceremony included Queen Emma of the Netherlands, the Queen of Würtemberg, the Hereditary Prince and Princess Frederick of Wied and Princess Louise of Wied, the Prince and Princess of Waldeck-Pyrmont, Princess Alexandra of Schaumburg-Lippe, the Prince and Princess of Bentheim Weinfurth. The wedding took place at half-past twelve, the Archbishop of Canterbury and other ecclesiastics officiating. The bride was conducted to the altar by her brother, the Duke of Saxe-Coburg and Gotha, and the King, who wore Field-marshal's uniform, gave her away. The five bridesmaids were Princess Margaret and Princess Victoria of Connaught, Princess Mary of Wales, Princess Mary of Teck, and Princess Helen of Waldeck-Pyrmont. The first part of the honeymoon will be spent at Brocket Hall, Hatfield, the seat of Lord Mount Stephen, and the remainder at Cannes.

### The Wedding Presents.

THE most notable of the nearly 300 of wedding presents to the Princess Alice of Albany were a magnificent tiara of diamonds and pearls by the King and Queen; a collar of diamonds by the Prince and Princess of Wales; a suite of Chippendale furniture by the Duke and Duchess of Connaught; a collar of brilliants of great beauty, in a light and open design of bows and festoons, a joint gift by Princess Louise Duchess of Fife, Princess Victoria, Prince and Princess Charles of Denmark, the Duke and Duchess of Teck, Prince Francis of Teck, and the Duke of Fife. The Duchess of Albany gave her daughter a silver-gilt tea service. The bridegroom gave his bride a superb tiara of diamond wheat-ears and a rivière of similar stones, the jewels having belonged to his mother, the late Princess Mary, Duchess of Teck; a sapphire and diamond ring, and a miniature of himself. The Duchess Marie of Saxe-Coburg and Gotha, the Crown Princess of Roumania, Princess Beatrice of Saxe-Coburg, and the ex-Grand Duchess of Hesse gave an exquisite brooch formed of two fine star sapphires set in a double loop of brilliants; Princess Christian of Schleswig-Holstein and Princess Victoria a glass claret jug in the shape of a duck, with chased silver head and feet; Princess Henry of Battenberg and her sons, four silver sauce boats and ladles; Princess Eugénie, a pierced and repoussé gold rose bowl, fitted with an emerald glass lining; Princess Victor Hohenlohe and the Countess Gleichen, a silver sugar bowl. The gift of the town of Kingston-on-Thames to the bride and bridegroom was a miniature single brougham of latest design, fitted with the electric light, and painted in green.

### Royal Visit to an Ice Carnival.

THE King and Queen, accompanied by the Prince and Princess of Wales, and other members of the Royal family, were present last week at an ice carnival, held at the National Skating Palace, Argyll Street, in aid of the funds of the Union Jack Club. The Royal party arrived at 10.30, and remained for an hour watching the skaters. There was fancy skating by several ladies and gentlemen, followed by a special display by a large company in fancy costumes. Special amusement was caused by two skaters who pulled round a small trolly on which were mounted two dolls representing Japan and China. Amongst the numerous guests who were present were Mrs. George Cornwallis West and Miss Ethel McCaul. The latter presided over the Army and Navy stall, where were exhibited specimens of woodwork, all of which were made from old battleships, including Nelson's flagship *Foudroyant*.

### War between Russia and Japan.

IT was officially announced on Monday that, at the instance of Japan, diplomatic relations between Russia and Japan had been broken off. The Japanese Minister at St. Petersburg was on Saturday instructed to demand his passports, and subsequently the Russian Legation left Tokio, the capital of Japan. All the Japanese residents in Neuchwang, Port Arthur, Mukden, and other parts of Manchuria have been ordered to leave, and the Japanese in the north of Korea have been advised to withdraw as far as Wousan and Ping-Yung. With regard to the cause of the rupture, the Russian Government affirm that the action of the Japanese Government in not waiting for the reply to their last despatch before they broke off negotiations imposes on Japan the entire responsibility of the consequences. On the other hand, the Japanese Government state that, having waited patiently for three weeks, they could wait no longer, especially as during that period Russia had been preparing to send troops to the Korean frontier. Japan opened hostilities at midnight on Monday by attacking, by torpedo-boats, the Russian squadron in the outer roads at Port Arthur, and succeeding in "damaging" two fine battleships and a second-class cruiser. A great naval battle, in which two Russian warships were sunk, has since been reported. Port Arthur has also been bombarded and damage done to a Russian battleship and three cruisers.

### Great Fire at Baltimore.

A FEARFUL conflagration broke out on Sunday in Baltimore. The first sign of the disaster was apparent at half-past ten in the morning. It was speedily followed by a series of explosions, and the frightened worshippers at the churches rushed out into the streets. It was soon seen that the fire had obtained a strong hold, and firemen were procured from Philadelphia, Washington, and New York, as well as police, physicians, and nurses. The fire engines and hose carts were conveyed by special trains. As nothing could be done to extinguish the flames it was decided to blow up several large edifices with dynamite so as to create a chasm, but though tons were used, in every case the walls stood firm, and the object was not achieved. Three-quarters of a mile of buildings have been burnt, the entire business quarter of Baltimore, and the financial loss will be as great as, if not greater than, at the fire in Chicago in 1871.

### The Release of Mrs. Maybrick.

IT was announced by the Home Secretary last week that Mrs. Maybrick had been granted a license under the Penal Servitude Acts, and is at present in a home, which she will be allowed to leave towards the end of the summer. On August 14th, 1889, Florence Elizabeth Maybrick was sentenced to death. She was convicted of poisoning her husband, Mr. James Maybrick, of Aigburth, Liverpool, a brother of the well-known singer and composer, Michael Maybrick, known also as Stephen Adams. Subsequently, Sir Charles Russell, who defended her with great skill, was largely instrumental in obtaining the reduction of the sentence to that of penal servitude for life. During most of her incarceration in Aylesbury Prison, Mrs. Maybrick worked in the laundry, but she was afterwards assigned the task of looking after the food arrangements and cleaning the utensils used at meals.

### A Book and its Story.

MRS. MEADE'S NEW NOVEL.*

Mrs. L. T. Meade maintains her popularity, as a voluminous writer of wholesome fiction, and her latest novel, "Nurse Charlotte," is no exception to many excellent predecessors of the same class. Girls who are self-centred should read it, for it is an object lesson in the power that lies in self-forgetfulness to influence the lives of others and their own for good. Perhaps the part of the story that will be least convincing to those knowing anything of hospital routine, is that which deals with the heroine as a probationer at St. Christopher's. But fiction does not pretend to be fact, and the fancy picture presented is only intended to be taken as such, probably, and a necessary adjunct to the development of the story.

Nurse Charlotte was the eldest daughter of a large family. Her parents came of good stock. Her father, a scholar of Baliol, had spent his life and his income in fruitless efforts to sustain his reputation as a man of letters. The story opens when burdened with debt, an invalid wife, and a hopeless outlook, he yet refuses to face in a practical way the future which he had brought upon his family by a policy of *laissez aller* and indifference to everything except a false estimate of his literary powers. Charlotte and her home environment are described as follows:—"Charlotte Home was a small girl with large grey eyes, black eyelashes, and a piquant, somewhat pale face. Her hair was dark brown and grew low on her forehead. When this story about her and her adventures, her noble efforts, her struggles to put wrong right, and her rare unselfishness comes to be written, she was exactly twenty-three years of age. From her earliest years Charlotte had had a burden to carry. She was the eldest child and she and poverty had made acquaintance from her birth. They were firm friends now and firm allies. She had discovered the way to take the sting out of poverty and to make the best of life." Charlotte's education had not been up to the modern standard, perhaps, but she was learned in all the gentler arts essential to a womanly nature. She had taught herself many of them, and could turn her hand to anything necessary to the comfort of her home, and those in it. "She could smooth the sick pillow and sit up at night with a fractious invalid and she could manage to do these things without any of the sunshine leaving her face or the brightness her manner."

Things at home had come to a crisis. Charlotte had two sisters—Alice, who was twenty, and Dorothy nineteen, but until now, she had so entirely supplied the invalid mother's place to the family, that she had hardly realised that they were old enough to take their share of responsibility in domestic matters. After a conversation with her father, she tells him of the decision she has come to. She had with difficulty brought him back to everyday facts from the region of impossible fancies into which, as usual, his mind had wandered when drifting on to the inevitable theme of his own unappreciated literary efforts.

The interview took place in Mr. Home's study which was the best room in the house, the walls being literally lined with books. Mr. Home used to refer to books as his one extravagance.) Charlotte at last managed to introduce the subject in which she was most interested, as being the means by which she hoped to be able, ultimately, to help her family, and make herself independent of her parents pecuniarily. "Do let us sit down and talk; I am very anxious to say something." "Dear me, Charlotte, I hope it is nothing disagreeable. I have had such trouble and the disappointment has been so keen (in reference to rejected manuscripts), I don't think I ought to be worried further to-day. What is it you want to say? If you can postpone it I know you will." "But I can't. It is impossible. I want to speak to you about the future. Things can't go on as they are. Someone must help. I believe I could be taken as a probationer at St. Christopher's Hospital, and go through my course of nursing as quickly as possible, and when I am a fully-trained nurse I could earn money for you all."

"You a nurse?" said Mr. Home, holding up his hands in amazement. "What in the world do you mean? How are we to spare you? . . . I think you are very selfish." "Indeed—indeed I am not. I am thinking of you. Do you suppose that I should like to go from you all?" "But you would be no present help. It is the present help we want." "I know it is." Little Charlotte stood with her hand pressed to her side. She could not help her family in any substantial way for quite two years, perhaps even three, and in the meantime Poverty and that grim wolf Want were knocking at the door. At the end of the discussion Mr. Home made the following reflection on Charlotte's character: "Tiresome child that," thought Mr. Home, "but Charlotte has a lot of grit in her; she gets that from me. I wonder what my poor family would do without someone to think for them."

Charlotte had a friend living in the neighbourhood who was rich and influential. She had always taken a great interest in her little friend and Charlotte was sure of a welcome whenever she called on Mrs. Stanhope at the Grange. The Grange was an Elizabethan mansion on the outskirts of the pretty village of Amhurst, in which the Homes lived. She turned to Mrs. Stanhope in her distress. Creditors were becoming importunate in their demands for money, and a recent threat conveyed to Mr. Home in a letter from the lawyer of a cousin, Digby McIver, to whom he owed five hundred pounds, demanding immediate payment, or the alternative of being sold up, had hastened her intention of asking her help and advice.

Mrs. Stanhope's sympathy took the practical form of introducing Charlotte to an eccentric but philanthropic elderly lady, who at once took in the bearings of the situation and summed up her intentions with regard to it as follows. "Now, Charlotte Home, I wish to speak to you. I have not the slightest doubt that you will act in an exceedingly silly way. But I mean to do just what I like with regard to you and yours. I look upon myself, Charlotte Home, as a steward, who has to dispense the very considerable estate left me by my Master. Now don't be annoyed, or proud, or disturbed, or anything of the sort. My employer, or in other words, my Divine Master, has told me that I am to help you, Charlotte Home, and I mean to do it." With such liberality were Miss Beck's promises carried into effect that Charlotte was able to leave home comparatively free from monetary anxiety, and to enter on a course of training at St. Christopher's. Her subsequent difficulties, her experiences, her love romance, and other matters of interest, we trust our readers will follow for themselves in Mrs. Meade's story of "Nurse Charlotte." One remark of a hospital patient, in a ward at St. Christopher's is worth quoting. Nurses, like other people, are not exempt from criticism tendered often, as in the present instance, in a crude, but not unkindly, manner. "She don't seem like a nurse," said one poor woman. "Half of them nurses are hard and like machinery. But she's all life—a sort of angel, I call her."

* "Nurse Charlotte." By L. T. Meade. (John Long. 6s.)

### Notes and Queries.

**REGULATIONS.**

**The Editor is always willing to answer in this column, without any fee, all reasonable questions, as soon as possible.**

**But the following rules must be carefully observed :—**

1. **Every communication must be accompanied by the name and address of the writer.**
2. **The question must always bear upon nursing, directly or indirectly.**

**If an answer is required by letter a fee of half-a-crown must be enclosed with the note containing the inquiry.**

*Fever Training.*

(166) I am anxious to get six months' fever training, and I should be much obliged if you will tell me which are considered the best schools?—*M. G.*

The hospitals of the Metropolitan Asylums Board are good schools. Apply to the Clerk, Victoria Embankment, E.C.

*L.O.S.*

(167) "Florence" would be glad to know the cheapest and best way in which to gain the L.O.S. certificate

Queen Charlotte's, Marylebone Road, N.W.; the British Lying-in Hospital, Endell Street, W.C.; the City of London Lying-in Hospital, City Road, E.C.; the General Lying-in Hospital, York Road, S.E., all give cheap and excellent training. Or write to the Secretary of the London Obstetrical Society, 20 Hanover Square, W.

*Massage.*

(168) Will you kindly let me know if there is any school or hospital for massage which grants certificates recognised by the medical profession?—*I. J. C.*

Write to the Secretary, the Incorporated Society of Trained Masseuses, 12 Buckingham Street, Strand, W.C.

*Laundry.*

(169) What is the usual sum allowed per head per week in a general hospital of 25 beds? The staff for laundry consists of the matron, four nurses, and three servants. The patients are supposed to provide their own linen, but the clothes brought by their friends is often insufficient for bad cases, and sometimes it is unfit to use.—*E. A. M.*

In most institutions the sum is from 7d. to 9d. per head per week.

*District Nurse.*

(170) Can you kindly inform me if there is an institution in East Dulwich which provides a nurse for the sick poor free, or for a small payment?—*Anxious Brother.*

The East Dulwich Provident Dispensary provides a Queen's nurse. Address 193 Landell's Road, S E.

*Convalescent Home.*

(171) Can you tell me of a convalescent home, not infectious, for nurses at or near Bristol, where only a small payment is made? —*M. A. J.*

The Memorial Institute, Almondsbury, six miles from Bristol, might suit you. Bristol nurses enjoy a reduction in fees.

*Travelling Expenses.*

(172) I was engaged to come on trial as probationer to an institution. The matron has told me that I must leave at the end of the month as I am not suitable for this kind of work. I am receiving a salary at the rate of £15 per annum, but, as I have had to pay for my uniform, I have no money to pay my fare home, will you kindly tell me if I can claim my travelling expenses?—*M. C.*

It depends entirely upon the terms of your agreement.

*Registration.*

(173) 1. I hold a four years' certificate for general nursing from a London Hospital and the L.O.S. I have just returned from doing private work abroad and am anxious to register, will you kindly give me particulars and say if the registration is for midwives only?—*D. H.*

1. Registration applies to midwives only. The Secretary, the Central Midwives Board, 6 Suffolk Street, Pall Mall, S.W., will give you all particulars.

Where can I obtain information with regard to becoming a registered nurse?—*Nurse B.*

See reply to *D. H.*

*Skin. Mourning.*

(174) 1. Will you please tell me if there are any hospitals for skin diseases in London, and which is the best? 2. Who is the greatest authority on skin diseases in the United Kingdom? 3. What form of mourning is it possible for a nurse to wear when on duty?—*M. M. M.*

1. There are three hospitals for diseases of the skin in London. The Hospital for Diseases of the Skin, 52 Stamford Street, Blackfriars, S.E.; St. John's Hospital for Diseases of the Skin, 49 Leicester Square, W.C.; and the British Hospital for Skin Diseases, 29 Euston Road, King's Cross, N.W. 2. We do not give names. 3. It is not usual for nurses to wear mourning when on duty.

*Abroad.*

(175) I am a fully-trained nurse, speaking German slightly, and I am anxious to go to that country either as a private or a hospital nurse. Will you kindly tell me how I can best accomplish this?—*Nurse B.*

Advertise in a German paper through a London advertising agency.

*Victorian Order of Nurses.*

(176) Will you kindly supply me with the London address of, and information about, the Victorian Order of Nurses for Canada?—*Betty.*

There is no London address. You can obtain information of the Order from the Headquarters, 578 Somerset Street, Ottawa, Ontario.

*Home.*

(177) Will you kindly tell me if there is a home where a middle-class lad of 15 could be received? He is dumb through paralysis of the tongue, but hears well. He must earn his own living; his friends can afford to pay a small sum if required.—*Nurse B.*

The list in "Burdett's Hospitals and Charities" might assist you.

*Mental Nursing.*

(178) I should be much obliged if you will tell me the earliest age at which I could enter an asylum for training in mental nursing. I am 18. Could you advise me of a good book on mental nursing similar to "The Nursing Profession: How and Where to Train?"—*L. W. W.*

Write to the Registrar, the Medico-Psychological Association of Great Britain and Ireland, 11 Chandos Street, W. "Mental Nursing," by Dr. Harding, Scientific Press, 28 Southampton Street, Strand, is a good text-book, but there is no directory to the institutions.

*Military Nursing.*

(179) Can you tell me if, in order to obtain a post as staff nurse in a military hospital, it is necessary to be hospital trained? Would one who has been trained in a Poor Law infirmary be eligible?—*Alexandra.*

You would be eligible if your Poor Law infirmary is a recognised training school. Apply to the Matron-in-Chief, Queen Alexandra's Imperial Military Nursing Service, 68 Victoria Street, S.W.

Will you kindly tell me to whom I must apply for the regulations preparatory to entering the army nursing staff; also if nurses trained at provincial hospitals are qualified for that position?—*E. M. D.*

See reply to *Alexandra.*

*Birth Certificate.*

(180) Will you kindly tell me if I can go in for the L.O.S. examination without a birth certificate? I am 27 years of age, but my birth certificate cannot be found.—*Nurse H.*

A birth certificate is not required by the London Obstetrical Society.

*Hospital Training.*

(181) My daughter, after having served two years of her term of three years, is afraid that the value of her certificate will be impaired, as the institution at which she is being trained is reducing its staff and number of beds. Can you tell me of a hospital or infirmary where she could begin again at the age of 22?—*F. S.*

Consult "The Nursing Profession: How and Where to Train."

**Standard Nursing Manuals.**

"The Nursing Profession: How and Where to Train." 2s. net; 2s. 4d. post free.

"Nursing: Its Theory and Practice." (Revised Edition). 3s. 6d. post free.

"Elementary Anatomy and Surgery for Nurses." By William McAdam Eccles, M.D.Lond., M.B. 2s. 6d. post free.

"Elementary Physiology for Nurses." By C. F. Marshall, M.D., B.Sc., F.R.C.S. 2s. post free.

"Ophthalmic Nursing." By Sydney Stephenson, M.B., F.R.C.S., 3s. 6d. net; 3s. 10d. post free.

"Nursing in Diseases of the Throat, Nose, and Ear." By P. Macleod Yearsley, F.R.C.S.Eng., M.R.C.S. 2s. 6d. post free.

"Fevers and Infectious Diseases." 1s. post free.

### The Nurses' Bookshelf.

Manual of Massage. By M. A. Ellison. (London: Baillière, Tindall and Cox; Paris; Madrid. 126 pages. 50 illustrations.)

In this manual there are only 15 pages dedicated to massage itself, seven to the Weir-Mitchell treatment, and two to the Swedish system. The rest of the book is devoted to a very useful and well-illustrated instruction on simple anatomy and physiology. This last-named section, which occupies the larger part of the book, is extremely well written, and shows accurate and extensive knowledge. The little that is said about massage seems good and clear, but nothing new or striking appears among the pages. One would (perhaps erroneously) think the "Dont's" on page 94 superfluous, as nurses are usually women of common sense; however, simple and yet all-important matters are often forgotten.

The Lord of the Dark Red Star: being the story of the supernatural influences in the life of an Italian despot of the thirteenth century. By Eugene Lee Hamilton. (The Walter Scott Publishing Company, Limited, London and Newcastle-on-Tyne. Price 6s. 1903.)

Mr. Eugene Hamilton has worked hard, and with no little success, to bring before us the six-centuries-old portrait of the dread tyrant Ezzelino, or Ezelin, steeped in that terrible madness or "blood fury," as it has been aptly named, which seized upon the arbitrary despot in Italian mediæval times. It is not to be wondered at that the horror-stricken contemporaries looked upon him and his like as physical as well as spiritual sons of Satan. There is a curious family likeness between the immortal Vathek's abominable mother Carathis and the equally iniquitous Adalhita, Ezelin's mother, though the books are written from very different points of view. The inimitable humour and pungent satire of Beckford's masterpiece is replaced in Mr. Lee Hamilton's story by an almost unrelieved gloom and misery, while he has too great a tendency throughout to "want to make your flesh creep," and, as it were, "to pile up the agony." Ezelin's descent into Hell is described with some originality, and it would be difficult not to plagiarise unconsciously a little from the great Italian poet who so long has held undisputed sway in that journey's portrayal. The influence of Dante's age and works is obvious throughout the work, as, for example, the romantic, unselfish love of Lionello and Hermann for Gisla and Selvaggia, illustrating the Troubadour spirit of devotion to a feminine ideal without hope of reward or requital, such as was Dante's love for Beatrice. Again, in Lionella's dream of his lost Gisla in Paradise "it appeared to him that as she passed she turned her head and fixed upon him a look of unspeakable tenderness," which recalls Beatrice's first salutation to Dante (*vita nuova*) and the profound effect her gaze ever had upon him (Purgatorio, canto xxxi.). In the Monk's Curse (p. 44) "the stream of boiling gore whose terrific billows shall toss round you when the cup of all your crimes is full" is the river of blood in the *Inferno* (canto xii., 46–48) "in which boils everyone who by violence injures others" (*vide* "Temple Classics" Edition, p. 125). The passages concerning the early attachment of Lionello and Gisla, and their pathetic travels in the children's crusade, are written in pleasing contrast to the otherwise lurid character of this work, and are fraught with sympathy and insight, as is also the awakening of maternal passion in Selvaggia's breast. A little more restraint, reserve and simplicity in the style and diction, would render the story none the less impressive. Passages such as "the sun was sinking in a rosy bed of quilted cloud" (p. 90), "darkening the darkness" (p. 233), and "to bound like bounding bales" only serve to weaken the effect they are desired to produce on the reader. The interest is, however, well maintained up to the dramatic ending, and poetical justice is fully satisfied by the death of the arch-tyrant and inhuman miscreant Ezelin.

### For Reading to the Sick.

"PRAY WITHOUT CEASING."

Or, rather, help us, Lord, to choose the good,
To pray for nought, to seek to none, but Thee,
Nor by "our daily bread" mean common food,
Nor say, "From this world's evil, set us free":
Teach us to love, with Christ, our sole true bliss,
Else, though in Christ's own words, we surely pray amiss!

*Keble.*

Pray! though the gift you ask for
May never comfort your fears,
May never repay your pleading,—
Yet pray, and with hopeful tears!
An answer,—not that you long for,
But diviner,—will come some day;
Your eyes are too dim to see it,
Yet strive and wait and pray!

*A. Procter.*

No duty is more insisted on in holy Scripture than that of prayer, nor is there anything more wonderful than God's love of prayer. "Pray without ceasing: be instant in prayer." "Ask and you shall receive." But you say you cannot pray much, especially when weak and in pain. Remember, then, the paths of prayer. Prayer is an offering to God, rising like clouds of incense before His throne above. And that offering goes by the path of words and thoughts, by the path of toil, and also by the path of pain and weariness. One is as good as another, provided it be pointed out by the finger of God as the path by which He desires your offering to ascend. He is very near to you in your sickness, and welcomes all the glory you can give Him by your pain. He writes it all down in the books of life as a reason for a hundredfold reward some day. "Fear not, therefore."

Often think of the Fatherhood of God, and gain courage, resignation, patience and strength from the thought. Often, in your hours of weakness, say the simplest and best of all prayers, the "Our Father." Be not solicitous; the very hairs of your head are all numbered; your Father knoweth of what you stand in need.

Probably no period of your life has given more glory to God, or been so full of true prayer, as the days of your sickness. It is a penitential prayer indeed that rises from your bed to God, but your prayer of pain is heard by Him and will in His good time be answered.

Let your petitions be made known to God, for the Lord will be entreated in favour of His servants; and it is your part to rest content, knowing that at the time of prayer the Lord is nigh unto us, His hands full of gifts, and that he grants to each one what is good in His sight.

"Lord, teach us thus ever to pray; teach us to go with confidence to the throne of grace, that we may obtain mercy and find grace to help in time of need."—*Anon.*

He may not in His mercy grant
The very thing you ask or want,—
Still, in this comfort rest—
His Power, His Wisdom, and His Love
In bright and boundless concord move,
He'll do the thing that's best.

*S. B. Monsell.*

